# Supplementary material for: A single cluster of RNA Polymerase II molecules is stably associated with active genes
Source: Nat Commun. 2026 Mar 20;17:2580. doi: 10.1038/s41467-026-70775-8 (PMC13004947; doi:10.1038/s41467-026-70775-8)
Supplement: Supplementary file 2 — Description of Additional Supplementary Files [file 41467_2026_70775_MOESM2_ESM.pdf]

Description of Additional Supplementary Files

Movie S1: Example of single molecule tracking in a live embryo of (left) an mEos3.2-RPB1, (middle) H2B-mEos3.2, and (right) NLS-mEos4.0. In all cases, the movies shown here are over a 3 second period (i.e. 300 frames at 10 ms). All movies are sped up to 8 fps.

Movie S2: Movie of an embryo showing hunchback MS2 spots in nc14 after vehicle injection in nc13. Each frame is the maximum intensity projection of a z-stack composed of 64 slices with 300 nm slice thickness and exposure time of 60 msec. The time interval between each stack is 10 sec. Movie is sped up to 8 fps.

Movie S3: Embryo in nc14 showing that hunchback MS2 spots disappear after α-amanitin injection in nc13. Each frame is the maximum intensity projection of a z-stack composed of 64 slices with 300 nm slice thickness and exposure time of 60 msec. The time interval between each stack is 10 sec. Movie is sped up to 8 fps.

Movie S4: Embryo in nc14 showing that hunchback MS2 spots disappear after triptolide injection in nc13. Each frame is the maximum intensity projection of a z-stack composed of 64 slices with 300 nm slice thickness and exposure time of 60 msec. The time interval between each stack is 10 sec. Movie is sped up to 8 fps.

Movie S5: Volumetric imaging of eGFP-RPB1 in a vehicle injected embryo from nc10 till the end of nc13. This movie highlights the RPB1 clusters observed across the nuclear cycles. Each frame is the maximum intensity projection of a z-stack composed of 64 slices with 300 nm slice thickness and exposure time of 60 msec. The time interval between each stack is 10 sec. Movie is sped up to 8 fps.

Movie S6: Volumetric imaging of eGFP-RPB1 in an α-amanitin injected embryo in nc14. The embryo was injected in nc12 and imaged from nc13 onwards. Each frame is the maximum intensity projection of a z-stack composed of 64 slices with 300 nm slice thickness and exposure time of 60 msec. The time interval between each stack is 10 sec. Movie is sped up to 8 fps.

Movie S7: Volumetric imaging of eGFP-RPB1 in a triptolide injected embryo in nc14. The embryo was injected in nc12 and imaged from nc13 onwards. Each frame is the maximum intensity projection of a z-stack composed of 64 slices with 300 nm slice thickness and exposure time of 60 msec. The time interval between each stack is 10 sec. Movie is sped up to 8 fps.

Movie S8: Example of tracked clusters in a vehicle injected embryo (single z-slice) for determination of average lifetime of clusters. Yellow circles indicate tracked clusters. Scale bar is 1 µm.

Movie S9: Example of tracked clusters in an α-amanitin injected embryo (single z-slice) for determination of average lifetime of clusters. Yellow circles indicate tracked clusters. Scale bar is 1 µm.

Movie S10: Example of tracked clusters in a triptolide injected embryo (single z-slice) for determination of average lifetime of clusters. Yellow circles indicate tracked clusters. Scale bar is 1 µm.

Movie S11: Examples of RNAPII clusters associating with a spot of transcription for each reporter gene in nc14. RNAPII is shown in the grey channel and MS2 spot is shown in the magenta channel. Scale bar is 1 µm for each movie. Time interval is 9 sec. Movies are sped up to 8 frames per second.

Movie S12: Examples of simulated movies showing cluster strength at an active gene increases with increasing k_{on} (from left to right). Time interval is 9 sec. Movies are sped up to 10 frames per second.

Movie S13: Examples of simulated movies showing cluster strength at an active gene increases with increasing promoter on-time and gene length. Time interval is 9 sec. Movies are sped up to 10 frames per second.
