# Supplementary material for: A single cluster of RNA Polymerase II molecules is stably associated with active genes
Source: Nat Commun. 2026 Mar 20;17:2580. doi: 10.1038/s41467-026-70775-8 (PMC13004947; doi:10.1038/s41467-026-70775-8)
Supplement: Supplementary file 16 — Reporting Summary [file 41467_2026_70775_MOESM16_ESM.pdf]

nature portfolio

Corresponding author(s): Apratim Mukherjee, Manya Kapoor, Kareena Shankta, Samantha Fallacaro, Raymond D. Carter, Gabriela Hayward-Lara, Puttachai Ratchasanmuang, Yara I. Haloush, Mustafa Mir

Last updated by author(s): Dec 27, 2025

Reporting Summary

Nature Portfolio wishes to improve the reproducibility of the work that we publish. This form provides structure for consistency and transparency in reporting. For further information on Nature Portfolio policies, see our Editorial Policies and the Editorial Policy Checklist.

Statistics

For all statistical analyses, confirm that the following items are present in the figure legend, table legend, main text, or Methods section.

n/a	Confirmed	
☐	☒	The exact sample size (n) for each experimental group/condition, given as a discrete number and unit of measurement
☐	☒	A statement on whether measurements were taken from distinct samples or whether the same sample was measured repeatedly
☐	☒	The statistical test(s) used AND whether they are one- or two-sided<br>*Only common tests should be described solely by name; describe more complex techniques in the Methods section.*
☐	☒	A description of all covariates tested
☐	☒	A description of any assumptions or corrections, such as tests of normality and adjustment for multiple comparisons
☐	☒	A full description of the statistical parameters including central tendency (e.g. means) or other basic estimates (e.g. regression coefficient) AND variation (e.g. standard deviation) or associated estimates of uncertainty (e.g. confidence intervals)
☐	☒	For null hypothesis testing, the test statistic (e.g. F, t, r) with confidence intervals, effect sizes, degrees of freedom and P value noted<br>*Give P values as exact values whenever suitable.*
☒	☐	For Bayesian analysis, information on the choice of priors and Markov chain Monte Carlo settings
☒	☐	For hierarchical and complex designs, identification of the appropriate level for tests and full reporting of outcomes
☒	☐	Estimates of effect sizes (e.g. Cohen's d, Pearson's r), indicating how they were calculated

Our web collection on statistics for biologists contains articles on many of the points above.

Software and code

Policy information about availability of computer code

Data collection	Custom Labview software was used for data collection (version number 4.09325 01-01-2022 developed at Janelia Research Campus)
Data analysis	The following softwares were used for data analysis:<br><br>(i) Python (version 3.7.4)<br>(ii) ImageJ (version 1.54p)<br>(iii) Quot (for single-molecule tracking; version v2)

For manuscripts utilizing custom algorithms or software that are central to the research but not yet described in published literature, software must be made available to editors and reviewers. We strongly encourage code deposition in a community repository (e.g. GitHub). See the Nature Portfolio guidelines for submitting code & software for further information.

Data

Policy information about availability of data

All manuscripts must include a data availability statement. This statement should provide the following information, where applicable:

- Accession codes, unique identifiers, or web links for publicly available datasets
- A description of any restrictions on data availability
- For clinical datasets or third party data, please ensure that the statement adheres to our policy

Data and code have been uploaded to Zenodo and can be accessed at 10.5281/zenodo.18174339

Research involving human participants, their data, or biological material

Policy information about studies with human participants or human data. See also policy information about sex, gender (identity/presentation), and sexual orientation and race, ethnicity and racism.

Reporting on sex and gender	N/A
Reporting on race, ethnicity, or other socially relevant groupings	N/A
Population characteristics	N/A
Recruitment	N/A
Ethics oversight	N/A

Note that full information on the approval of the study protocol must also be provided in the manuscript.

Field-specific reporting

Please select the one below that is the best fit for your research. If you are not sure, read the appropriate sections before making your selection.

☒ Life sciences ☐ Behavioural & social sciences ☐ Ecological, evolutionary & environmental sciences

For a reference copy of the document with all sections, see nature.com/documents/nr-reporting-summary-flat.pdf

Life sciences study design

All studies must disclose on these points even when the disclosure is negative.

Sample size	No formal statistical power calculation was performed prior to data collection. Instead, we aimed to acquire a sufficiently large number of single-molecule trajectories (>100,000 per condition across 3 or more embryos) based on our prior experience with single-molecule tracking. For the volumetric imaging, we aimed to acquire a minimum of 3 independent embryos per developmental stage and drug condition to ensure statistical confidence in the measured parameters.
Data exclusions	No data was excluded from the study.
Replication	Embryos were staged according to the nuclear cycle (well described in the literature) rather than randomly assigned to groups. Because each nuclear cycle or stage is an intrinsic developmental state, no random allocation was possible. Instead, we collected data from at least three independent embryos per stage and per drug injection to ensure biological replicates and to account for inter-embryo variability.
Randomization	Embryos were staged according to the nuclear cycle (well described in the literature) rather than randomly assigned to groups. Because each nuclear cycle or stage is an intrinsic developmental state, no random allocation was possible. Instead, we collected data from at least three independent embryos per stage and per drug injection to ensure biological replicates and to account for inter-embryo variability.
Blinding	It was not feasible to blind investigators to the developmental stage of each embryo because these stages are morphologically distinct and inherently obvious during imaging. However, single-molecule tracking, volumetric imaging and subsequent associated data analyses were all performed using our in-house automated pipelines developed on Python. The use of these pipelines for all samples minimizes the potential for investigator bias.

Reporting for specific materials, systems and methods

We require information from authors about some types of materials, experimental systems and methods used in many studies. Here, indicate whether each material, system or method listed is relevant to your study. If you are not sure if a list item applies to your research, read the appropriate section before selecting a response.

Materials & experimental systems

n/a	Involved in the study	
☐	☒	Antibodies
☒	☐	Eukaryotic cell lines
☒	☐	Palaeontology and archaeology
☐	☒	Animals and other organisms
☒	☐	Clinical data
☒	☐	Dual use research of concern
☒	☐	Plants

Methods

n/a	Involved in the study	
☒	☐	ChIP-seq
☒	☐	Flow cytometry
☒	☐	MRI-based neuroimaging

Antibodies

Antibodies used

Anti-RNA polymerase II CTD repeat YSPTSPS (phospho S5) antibody (ab5131), Recombinant Anti-beta Tubulin antibody (ab179513), RPB1 CTD (4H8) Mouse mAb (2629T), and α-Tubulin Antibody (2144).

Validation

The AbCam antibodies have been validated for immunocytochemistry/immunofluorescence (ICC/IF), western blotting (WB), chromatin immunoprecipitation (ChIP), immunoprecipitation (IP), and immunohistochemistry (IHC) as per their website.
The antibodies from Cell Signaling Technologies have similarly been validated for western blotting (WB), and immunohistochemistry as per their website.

Animals and other research organisms

Policy information about studies involving animals; ARRIVE guidelines recommended for reporting animal research, and Sex and Gender in Research

Laboratory animals

The details for the fly lines used in this study are reported in the Key Resources section of the manuscript. These details are reproduced below:
RPB1-LL-eGFP (The line was made using CRISPRCas9 system. The construct was
made with 1x-FlagTag at the Nterminal of the eGFP. The eGFP is
linked to the RPB1 with the long linker
sequence:
SGDSGVYKTRAQASNSAVDGTAGP
GSTGSS.)
His2B-eGFP (Fly line with an H2B-eGFP transgene
inserted on chromosome 3.
Transgene is expressed ubiquitously
under the control of a synthetic tubulin
promoter.)
RPB1-LL-mEos3.2 (The line was made using CRISPRCas9 system. The construct was
made with 1x-FlagTag at the Nterminal of the mEos. The mEos is
linked to the RPB1 with the long linker sequence:
SGDSGVYKTRAQASNSAVDGTAGP
GSTGSS.)
His2B-mEos3.2 (Fly line with an H2BmEos3.2
transgene inserted on chromosome 3.
Transgene is expressed ubiquitously
under the control of a synthetic tubulin
promoter.)
NLS lines (Fly line with an NLS-mEos3.2
transgene inserted on chromosome 3.
Transgene is expressed ubiquitously
under the control of a synthetic tubulin
promoter.
NLS11: AAAKRSWSMAF was used).
UbxP-phiC31-P3RFP (Fly line with the endogenous Ubx promoter and Exon 1 replaced by an attP P3-RFP cassette. For RMCE at the endogenous Ubx promoter.)
Inverted Ubx-ParS2-MS2 (Fly line generated by RMCE of UbxP-phiC31-P3RFP. Contains a ParS2-MS2-UbxP_exon1 cassette inserted in the reverse direction.)
ParB-mCherry, eve-MS2, sna-MS2, hb-MS2 lines obtained from Garcia Lab at UC Berkeley. sog-MS2 line obtained from Lim lab at UPenn.

Wild animals

N/A

Reporting on sex

N/A

Field-collected samples

N/A

Ethics oversight	N/A

Note that full information on the approval of the study protocol must also be provided in the manuscript.

Plants

Seed stocks	N/A
Novel plant genotypes	N/A
Authentication	N/A
